# A clinical perspective on imaging in juvenile idiopathic arthritis

**DOI:** 10.1007/s00247-023-05815-2

**Published:** 2023-11-28

**Authors:** Maria Tarsia, Mojca Zajc Avramovič, Ana Gazikalović, Damjana Ključevšek, Tadej Avčin

**Affiliations:** 1 Clinical Paediatrics, Department of Molecular Medicine and Development, University of Siena, Siena, Italy; 2 Department of Allergology, Rheumatology and Clinical Immunology, University Children’s Hospital, University Medical Centre Ljubljana, Bohoričeva 20 SI-1525, 1000 Ljubljana, Slovenia; 3 Department of Pediatrics, Faculty of Medicine, University of Ljubljana, Ljubljana, Slovenia; 4 Department of Radiology, University Children’s Hospital, University Medical Centre Ljubljana, Bohoričeva ulica 20, 1000 Ljubljana, Slovenia

**Keywords:** Children, Conventional radiography, Imaging, Juvenile idiopathic arthritis, Magnetic resonance imaging, Ultrasound

## Abstract

In recent years, imaging has become increasingly important to confirm diagnosis, monitor disease activity, and predict disease course and outcome in children with juvenile idiopathic arthritis (JIA). Over the past few decades, great efforts have been made to improve the quality of diagnostic imaging and to reach a consensus on which methods and scoring systems to use. However, there are still some critical issues, and the diagnosis, course, and management of JIA are closely related to clinical assessment. This review discusses the main indications for conventional radiography (XR), musculoskeletal ultrasound (US), and magnetic resonance imaging (MRI), while trying to maintain a clinical perspective. The diagnostic-therapeutic timing at which one or the other method should be used, depending on the disease/patient phenotype, will be assessed, considering the main advantages and disadvantages of each imaging modality according to the currently available literature. Some brief clinical case scenarios on the most frequently and severely involved joints in JIA are also presented.

**Graphical abstract:**

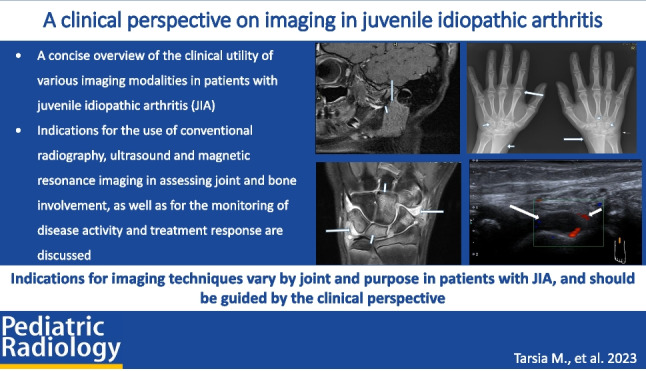

## Introduction

Juvenile idiopathic arthritis (JIA) is the most common chronic rheumatic disease of childhood [1]. It has been defined according to the American College of Rheumatology (ACR) criteria, which require an age less than 16 years at onset and persistence of arthritis for a period of at least 6 weeks, following the exclusion of alternative potential causes of arthritis [2]. The current International League of Associations for Rheumatology (ILAR) classification for JIA is based on the extent of disease in the first six months and recognizes seven disease categories, summarized in Table 1.

**Table 1:** International League of Associations for Rheumatology (ILAR) classification of juvenile idiopathic arthritis: categories, frequency, onset age distribution, and sex prevalence [1]

	Frequency^a^	Onset age	Sex ratio
Enthesitis-related arthritis	3–11%	Late childhood or adolescence	M>>F
Oligoarthritis	27–56%	Early childhood	F>>>>M
Psoriatic arthritis	2–11%	Biphasic distribution; early peak at 2–4 years and later peak at 9–11 years	F>M
Rheumatoid-factor-positive polyarthritis	2–7%	Late childhood or adolescence	F>>>M
Rheumatoid-factor-negative polyarthritis	11–28%	Biphasic distribution; early peak at 2–4 years and later peak at 6–12 years	F>>M
Systemic arthritis	4–17%	Throughout childhood	F=M
Undifferentiated arthritis	11–21%	–	–

^a^Reported frequencies refer to percentage of all juvenile idiopathic arthritis

*F* female, *M* male

Imaging plays a pivotal role in identifying the presence, severity, and extent of musculoskeletal involvement. It helps in monitoring disease complications, ruling out alternative diagnoses, and evaluating the response to treatment [3, 4].

Nevertheless, interpretation of skeletal imaging in children can be challenging due to the developing nature of the growing skeleton. It requires substantial experience and effort to effectively differentiate between healthy subjects and those with disease-related changes. Establishing reference standards and robust scoring systems tailored to the developmental stage of children remains an ongoing endeavor [5].

Although the European League Against Rheumatism (EULAR)/Pediatric Rheumatology European Society (PRES) imaging task force has recently highlighted the potential role of imaging in the diagnosis, progression, and monitoring of treatment efficacy in various aspects of JIA, the clinician’s primary focus remains the interweaving between an excellent clinical examination and the judicious use of laboratory and imaging techniques [6].

In this review, we aim to provide clarity on which patients and joint imaging can be used and at what time they should be used. We will outline the main indications for various imaging techniques depending on the joint and purpose, always keeping the clinical aspect in mind. We will also highlight the advantages and disadvantages associated with these imaging techniques.

## State of play on imaging in juvenile idiopathic arthritis

Considerable progress has been made in recent decades to improve the quality of diagnostic imaging and to reach a consensus on the methods and scoring systems to be used.

The latest OMERACT (Outcome Measures in Rheumatology Clinical Trials) Filter, uploaded in version 2.1, provided the tools to assess the quality of an imaging technique by evaluating the outcomes against the three “pillars” of truth, discrimination, and feasibility, as well as their ability to discriminate between different degrees of disease [7].

Over the past years, several international collaborative research groups on JIA imaging have emerged. In 2018, Nusman et al. [8] provided an overview of ongoing international initiatives, their focus, and imaging-related outcomes in JIA. Some study groups concentrate on all available imaging modalities (e.g., the ACR’s Pediatric Rheumatology Working Group or the EULAR-PRES Task Force) and encompass all joints. Other groups specialize in magnetic resonance imaging (MRI) (such as the OMERACT group, subdivided into three subgroups for small joints, large joints, and the temporomandibular joint [TMJ]) or in ultrasound (US) (e.g., the Childhood Arthritis and Rheumatology Research AllianceUS Group) [8].

## Experts’ opinion: critical points

The potential use of imaging studies in modern pediatric rheumatology practice, alongside its main challenges in JIA, remains controversial in both clinical practice and research. This ambiguity can be attributed to several factors, such as the absence of standardized imaging protocols specifically designed for pediatric populations and the difficulty associated with evaluating tissues that are still maturing and developing. While highlighting the higher sensitivity of musculoskeletal US and MRI in detecting inflammation in asymptomatic joints and their potential aid in early diagnosis, it is important to note that a significant challenge remains: the need for consensus on fundamental lesions and the standardization of imaging protocols [4, 9–11].

In addition, MRI should be optimized as a robust biomarker and outcome measure. Nevertheless, its application for treatment monitoring is still limited to clinical trials, as estimates of bone erosion and cartilage loss in children remain inherently imprecise. MRI is known to be constrained by several limitations, including cost, accessibility, and patient acceptance [12, 13].

Regarding the prognostic value of musculoskeletal US in predicting disease flare-up and planning therapeutic strategy, it remains unclear whether subclinical synovitis carries the risk of silent progression of joint damage and should influence the clinician’s decision to discontinue treatment [14].

Finally, clinical questions about the timing of imaging examinations, the selection of appropriate imaging modalities, and how many joints should be evaluated remain partially unresolved.

## When should imaging be used in juvenile idiopathic arthritis?

### Diagnosis

In adult patients, MRI and musculoskeletal US have been integrated into diagnostic algorithms to assess the extent of joint involvement, as outlined in the 2010 revised diagnostic criteria for rheumatoid arthritis, increasing diagnostic accuracy, particularly in early disease stages [15]. In addition, Duer-Jensen et al. [16] regarded MRI bone marrow edema and the combined synovitis and erosion pattern as valuable indicators for diagnosing rheumatoid arthritis.

Contrastingly, a specific imaging signature for JIA has not yet been described. Up to now, no study has specifically addressed the role of MRI in the diagnosis of JIA, although several papers have reported that MRI can assist physical examination in the early differentiation of childhood arthritis.

Even though the diagnosis of JIA remains one of exclusion, based solely on clinical criteria, imaging is increasingly being used to help to confirm the diagnosis.

Conventional radiography (XR) can reveal early-stage manifestations of the disease, including soft tissue swelling and periostitis. However, these findings are not exclusive to arthritis, and their sensitivity is very limited, especially in detecting early-stage disease [17].

Musculoskeletal US is of particular importance as it can detect subclinical synovitis and improve the classification of patients into different JIA subtypes [18].

MRI provides distinct advantages over clinical evaluation, particularly when assessing specific joints, due to its capability to image the TMJ and the axial skeleton, for which MRI serves as a reference method for detecting early changes [19–23].

### Initial confirmation of the clinical diagnosis and differential diagnoses

In cases where the history or clinical findings are inconclusive, XR, musculoskeletal US, or MRI may be used to improve the certainty or uncertainty of a JIA diagnosis beyond clinical features and to narrow the differential diagnosis [6].

**Fig. 1:**
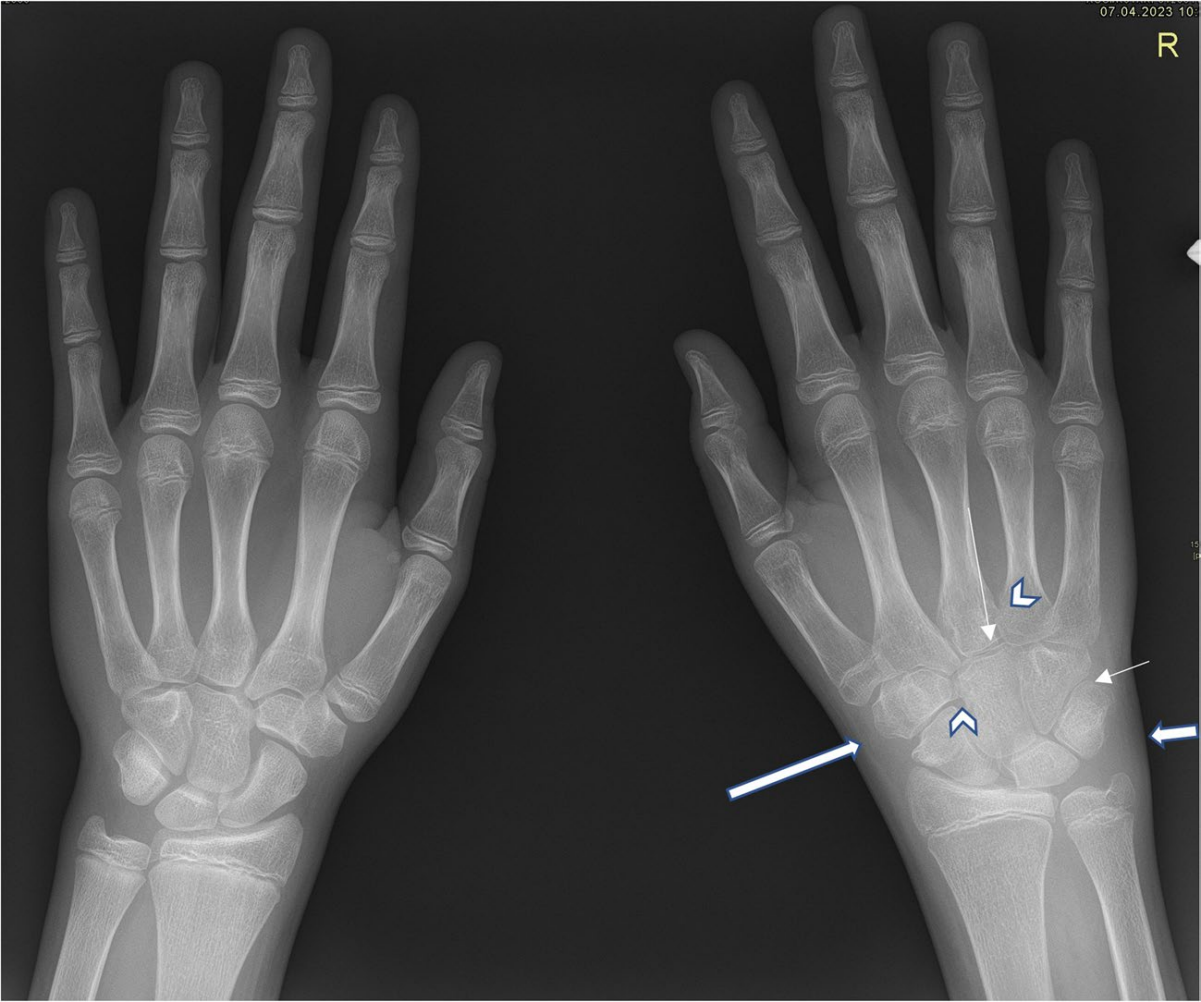
Anteroposterior radiograph of both hands in a 13.5-year-old boy with enthesitis-related (HLA B27 positive) juvenile idiopathic arthritis: general mild osteoporosis of the right hand (*long arrow*), soft tissue edema around the carpal bones on the right (*short arrow*), narrowed carpometacarpal joint spaces on the right (*long thin arrow*), discrepancy of the size of the carpal bones due to accelerated growth on the right (*short thin arrow*), suspicious erosions at the base of the second to fourth metacarpals and at os trapezoideum and os capitatum (*arrowheads*). Normal structure and morphology of the bones of the left hand

In the early stages of disease, XR has its place in excluding other bone pathology, such as trauma, tumors, avascular necrosis, bone dysplasia, osteomyelitis, and other bone marrow or hematological disorders affecting joints, such as leukemia or hemophilia [24, 25]. XR is crucial for the early detection of disease-related damage, detecting subclinical changes that may have occurred prior to diagnosis, such as soft tissue thickening, joint effusion, periarticular changes, and periarticular or diffuse osteoporosis (Fig. 1) [19].

Musculoskeletal US is valuable in identifying extra-articular causes of tissue swelling mimicking joint effusion [26] and in determining the site of inflammation by differentiating between synovial, tendinous, and entheses involvement [27–29].

**Fig. 2:**
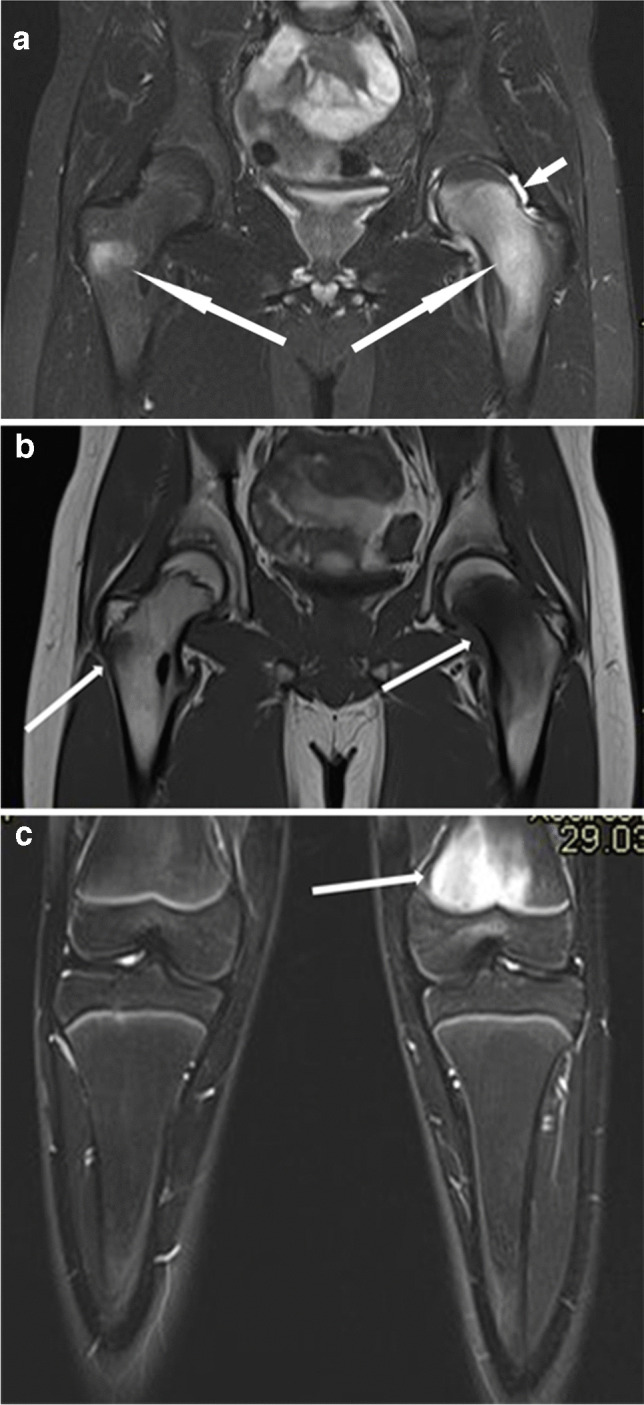
Magnetic resonance images in a 15-year-old boy with chronic recurrent multifocal osteomyelitis. **a**, **b** Coronal T2 turbo inversion recovery magnitude (TIRM) (**a**) and T1 (**b**) images of the hips show bone edema in the metaphysis and epiphysis of the left femur and at the base of the right greater trochanter (*long arrows*). There is a small reactive effusion in the left hip joint (*short arrow* in **a**). **c** Coronal T2 TIRM image shows bone edema in the distal metaphysis of the left femur (*arrow*)

The role of MRI remains undisputed in ruling out other joint or synovial pathologies that may mimic JIA (pigmented villonodular synovitis, hemangioma, synovial chondromatosis, lipoma arborescens, or chronic recurrent multifocal osteomyelitis (Fig. 2) [30, 31].

### Assessment of disease activity/remission and treatment efficacy

Many studies on rheumatoid arthritis have shown that residual synovitis is associated with a significant risk of disease relapse and progression of structural damage. MRI is increasingly being incorporated as an endpoint in numerous clinical trials aimed at evaluating the effectiveness of novel antirheumatic drugs for adult patients [32–35].

In JIA, multiple studies have been performed that have aimed to link imaging results with the ability to evaluate disease activity and treatment efficacy.

Literature articles from 2011 to 2013 have shown that the presence of US-detected synovial abnormalities, including power Doppler signals, does not predict subsequent synovitis flare in JIA patients in clinical remission. Interestingly, this contrasts with the findings in adults. Paradoxically, patients with persistent inactive disease had a higher frequency of power Doppler signals than those who experienced a disease flare [14, 36–38].

Subsequently, with improved US technology, it was demonstrated that musculoskeletal US abnormalities were shown to increase the risk of disease relapse in clinically inactive JIA patients by almost fourfold. This highlights musculoskeletal US as a valuable tool for stratifying the risk of disease relapse in patients with JIA in clinical remission [39, 40].

A recent study by Mazzoni et al. [12] found that subclinical synovitis and bone marrow edema detected on MRI in 65.5% and 46.7% of patients in their cohort, respectively, were the best predictors of disease relapse and joint deterioration, despite clinical remission. These findings have important implications for disease management [12].

Due to the lack of standardized measurements and techniques, imaging is not currently part of the composite measures of disease activity, which focus on clinician- and patient-reported assessments and inflammatory markers. Nevertheless, imaging is often used in clinical practice to guide decisions, especially for joints that are difficult to assess clinically (TMJ, sacroiliac joint (SIJ), cervical spine) [20–42].

## Which imaging modalities should be used in juvenile idiopathic arthritis from the clinical perspective?

### Indications for conventional radiology

XR remains the most readily available imaging modality for detecting and monitoring structural damage and growth abnormalities, but given the current emphasis on early intervention, the detection of pre-erosive joint changes has become a priority.

However, in cases of clinical uncertainty, XR plays a crucial role, excluding other differential diagnostic options such as traumatic or orthopedic diseases (fractures, osteochondral lesions), tumors, and infectious causes (osteomyelitis) [4, 6].

In 2018, the Task Force of the French Societies of Rheumatology, Radiology, and Paediatric Rheumatology, focusing on XR, attempted for the first time to provide pragmatic guidelines for daily practice specific for each non-systemic JIA subtype and for situations of particular interest [43], summarized in Table 2.

**Table 2:** Conventional radiography in juvenile idiopathic arthritis: joint recommendations from the French societies for rheumatology, radiology and pediatric rheumatology [41]

Acute monoarthritis: - XR of the involved joint should be performed in two perpendicular views to exclude a tumor, osteomyelitis, or hematological malignancy Comparative XR of the contralateral joint is unnecessary
Cervical spine: - Lateral XR of the cervical spine is only indicated if MRI is unavailable In the presence of neurological symptoms of spinal cord compression and neck pain, cervical MRI to be performed on an emergency basis
Enthesitis-related arthritis (ERA): - XR of the spine and hip joints are limited to the differential diagnosis - During the follow-up of axial ERA, XR may be considered (only for the hip joints), depending on the clinical course and availability of US and/or MRI XR is not recommended for multifocal enthesitis. If isolated enthesitis, XR can be considered as a tool for establishing the differential diagnosis (osteochondritis)
Hip joint: - Routine XR is not recommended in pJIA If XR of a symptomatic hip joint is performed, only a single view should be obtained (antero-posterior or frog leg view)
Oligoarticular JIA (oJIA): - Should be performed on affected joint(s) that remain symptomatic^a^ after 3 months (not routine diagnostic)^b^ - In extended oJIA, apply recommendations for pJIA
Polyarticular JIA (pJIA): - If RF/ACPA+ , routine XR of the wrists, hands, and forefeet strongly recommended at time of diagnosis—1 year after disease onset and at transition from pediatric to adult healthcare - If RF/ACPA -, XR to be performed only in case of adverse prognostic factors (early involvement of wrists, symmetric arthritis, distal, small-joint arthritis, elevated ESR/CRP, pre-existing radiographic abnormalities) - In symptomatic* disease longer than 3 months, XR can be repeated^b^
Temporomandibular joints: - If cross-sectional imaging is available, XR is not recommended

^a^Symptomatic joints are painful and/or swollen joints and/or joints that have restricted mobility

^b^The selection and timing of specific follow-up imaging techniques to further assess structurally damaged joints is guided by clinical considerations

*ERA* enthesitis-related arthritis, *JIA* juvenile idiopathic arthritis, *MRI* magnetic resonance imaging, *p* polyarticular, *o* oligoarticular, *US* ultrasound, *XR* conventional radiography

Interestingly, Weiss et al. [44] recently provided the first consensus-derived radiographic definition of sacroiliitis in skeletally immature adolescents as a criterion for classifying axial disease in juvenile spondyloarthritis when MRI is not available. Nevertheless, the use of XR in the diagnosis of sacroiliitis is discouraged.

In recent decades, new radiological scoring systems have been developed and adult radiological scores have been adapted for use in JIA. Their application in non-controlled JIA clinical trials has demonstrated that standardized assessment of radiological progression is feasible. This has led to the suggestion that semiquantitative measurement of radiological damage should also be considered when evaluating treatment efficacy in JIA [45–47].

**Fig. 3:**
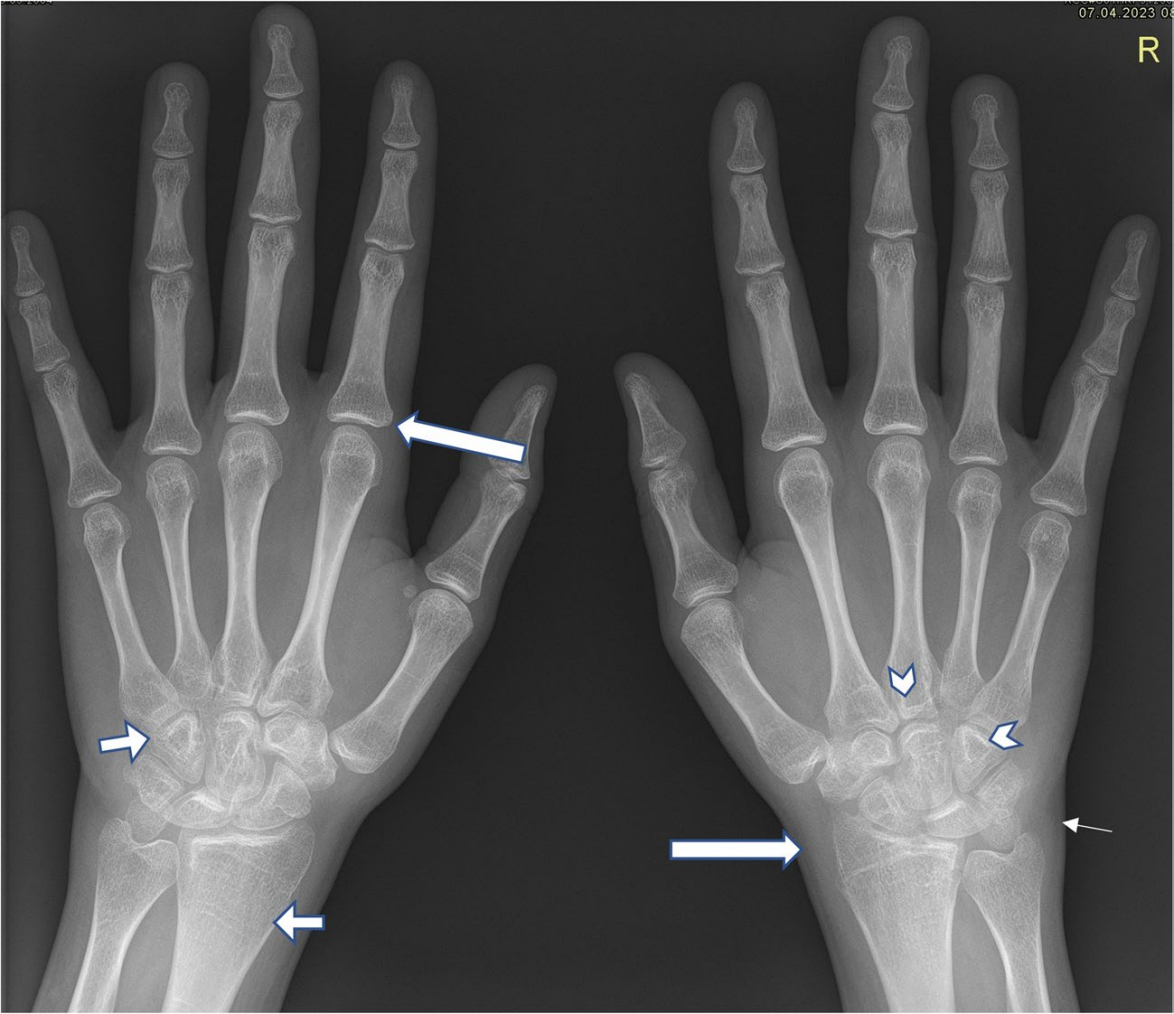
Anteroposterior radiographs of both hands in a 19-year-old young woman with a long history of aggressive seronegative polyarticular juvenile idiopathic arthritis, onset at the age of 7 years and with poor adherence to treatment. Images show mild bilateral periarticular osteoporosis (*long arrows*), growth arrest lines in the radial metaphysis and bone-in-bone appearance of carpal bones (*short arrows*), joint space narrowing of carpo-metacarpal joints and intercarpal bones, more pronounced on the right (*arrowheads*), and mild soft tissue edema around the right ulna (*thin arrow*)

In advanced stages of disease, XR allows visualization of late complications (erosions, ankylosis, subluxation or joint malalignment, enlarged epiphysis, premature growth plate fusion leading to limb length inequality, spinal deformities, muscle atrophy) (Fig. 3) [48–51].

In addition, XR has a historical role in assessing bone maturity and detecting bone age delay or progression, which in JIA may also help to distinguish where disease control is suboptimal or whether other factors are influencing growth retardation [52].

In summary, XR is a useful method for differential diagnosis in doubtful cases, evaluation of structural and morphological changes before diagnosis (indication based on clinical examination), and evaluation and monitoring of joint destruction and growth disorders.

### Indications for musculoskeletal ultrasound

Musculoskeletal US represents an easily accessible, clinically relevant routine examination in children with JIA.

Before US can be established as a valuable imaging modality, two significant challenges must be overcome: understanding age-related normal findings and standardizing the US protocol for different joints.

Numerous papers, including the 2018 OMERACT US in Paediatrics Working Group, have described the standardization of US examination for different joints, including physiological intra-articular vascularization, patient and joint position, and transducer placement for each examination approach [53–58].

Although these reports provide information to help differentiate between normal and pathological findings of joints in children, they currently serve as baseline information. Assessment of changes in US characteristics within an individual subject over time could potentially be more informative than a simple comparison with a cutoff value [56]. Another issue is the minimum and optimal set of joints that should be scanned for routine musculoskeletal US surveillance. Scanning all accessible joints is not feasible in routine practice, and different studies have tested different numbers of joints.

The reduced model with ten joints by Collado et al. [59] showed higher responsiveness to changes than the evaluation of a larger number of joints. Overall, these results suggest that an US assessment which focuses on a reduced number of joints and includes the sites that are most commonly affected in JIA may satisfactorily provide information about the overall burden of disease activity [59].

**Fig. 4:**
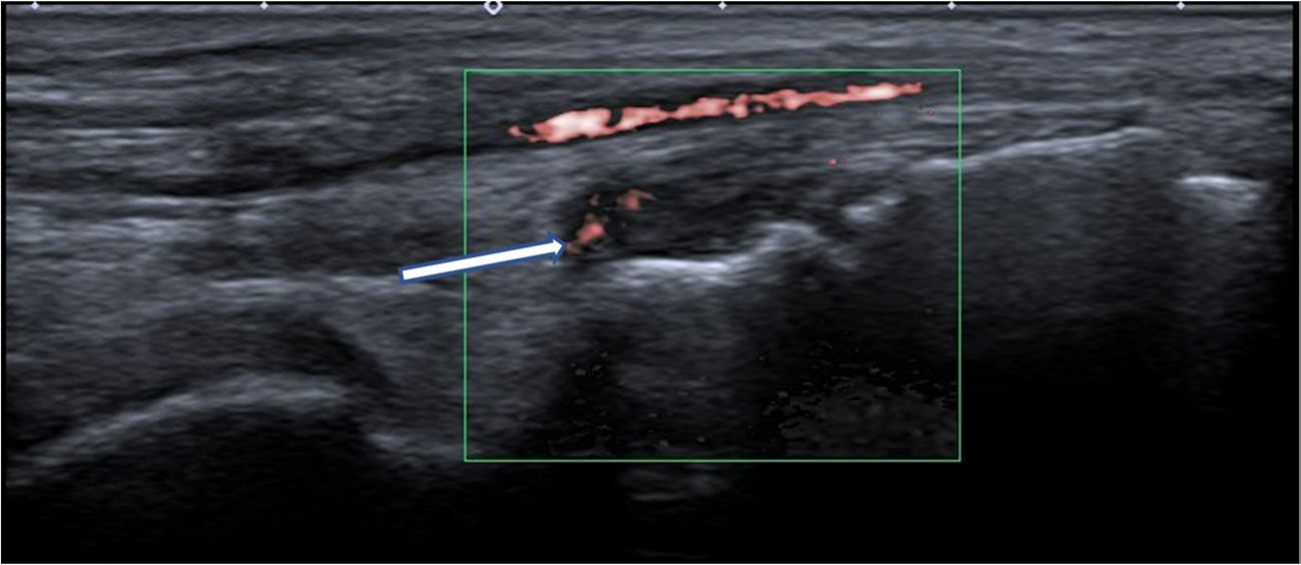
Power Doppler ultrasound of left talonavicular joint in sagittal projection in a 19-year-old young woman with a long history of aggressive seronegative polyarticular juvenile idiopathic arthritis, onset at the age of 7 years and with poor adherence to treatment (same patient as in Fig. 3) shows thickened and chronically altered synovium without a joint effusion or significant hyperemia (*arrow*)

**Fig. 5:**
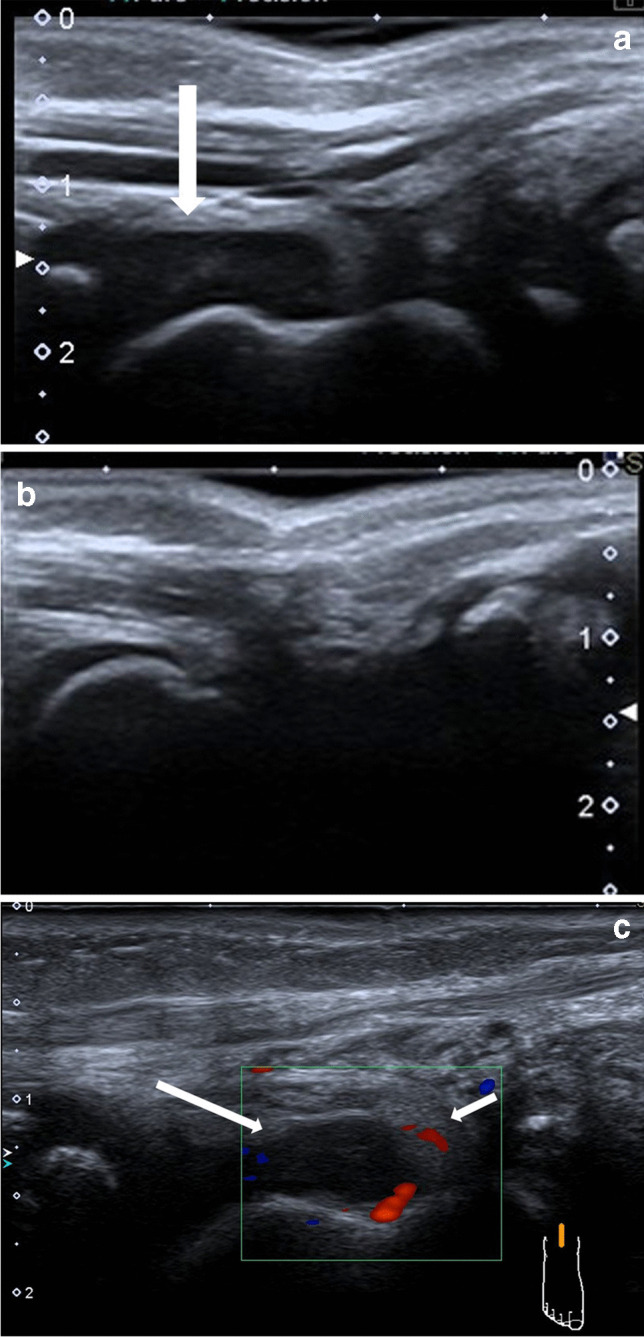
Ankle ultrasound of a 2-year-old girl with juvenile idiopathic arthritis (HLA B27 negative, anti-nuclear antibodies positive). **a**,** b** Sagittal projection of right (**a**) and left (**b**) ankle with effusion in the right side (*arrow*); (**c**) Sagittal power Doppler study of right ankle with effusion (*long arrow*), and thickened and hyperemic synovium (*short arrow*)—signs of synovitis

Joints most suitable for musculoskeletal US examination are the ankle, knee, hip, wrist, and small joints of the hands and feet (Figs. 4 and 5) [18]. Apart from the anatomical consideration and the challenge of clinical assessment of these joints, Magni-Manzoni et al. [60] have demonstrated a higher incidence of subclinical synovitis in the wrists, proximal interphalangeal (PIP) joints, subtalar joints, and ankles.

Although the role of US in assessing the axial skeleton remains limited, a recent study by Falsetti et al. [61] suggested the potential role of power Doppler ultrasound with spectral wave analysis as a screening method in children with suspected juvenile spondyloarthritis. They found higher power Doppler US scores at the SIJs in patients with a confirmed diagnosis of juvenile spondyloarthritis based on MRI diagnosis [61].

The most important clinical contribution of US is the identification and differentiation of synovitis, tenosynovitis, bursitis, and enthesitis.

In several studies, US has been shown to be superior to physical examination in the diagnosis of synovitis [26, 28, 37, 60, 62–68]. Nevertheless, it remains questionable whether physical examination or US can be more accurate in detecting joint inflammation. Indeed, confidence and competence in musculoskeletal examinations may be low, particularly in pediatrics. A systematic literature review on the assessment of synovitis in JIA by Collado et al. [69] on the assessment of synovitis in JIA highlighted key issues, such as small sample size, lack of MRI comparison, technical difficulties, and lack of a control score.

Enthesitis is the main feature of the JIA subgroup of enthesitis-related arthritis. Clinical recognition of enthesitis in children is challenging due to the particular distribution of fat, which can obscure anatomical landmarks, and the often-inadequate cooperation of very young children.

A number of studies confirm a higher sensitivity of musculoskeletal US compared to clinical examination in detecting enthesitis [70–73]. On the other hand, a recent systematic review indicated that the existing evidence suggests that there is no standardized US definition of enthesitis in children, and that discriminant validity has not been demonstrated [74].

The additional role of US is to monitor the response to treatment and the disease course. In this context, the importance of power Doppler US in addition to conventional US and the use of a standardized US scoring system seems crucial.

US-guided procedures are the next important application of US in JIA. They allow precise localization of inflammation and accurate needle placement in clinically difficult-to-assess or hard-to-reach sites, such as wrists, TMJs, hip, small joints of hands and feet, ankles, and tendons. This maximizes treatment efficacy while minimizing local side effects, such as subcutaneous atrophy or localized skin hypopigmentation [75–79]. Due to limited and conflicting data, certain critical aspects require further investigation, particularly for specific sites such as the TMJs [80].

In summary, musculoskeletal US is useful in daily practice to assess the presence and degree of inflammation in areas that are more difficult to assess clinically, such as the wrist, ankle, and foot joints. It can be used to guide intra-articular injections. Ideally, it should be performed by an experienced radiologist or rheumatologist.

### Indications for magnetic resonance imaging

**Fig. 6:**
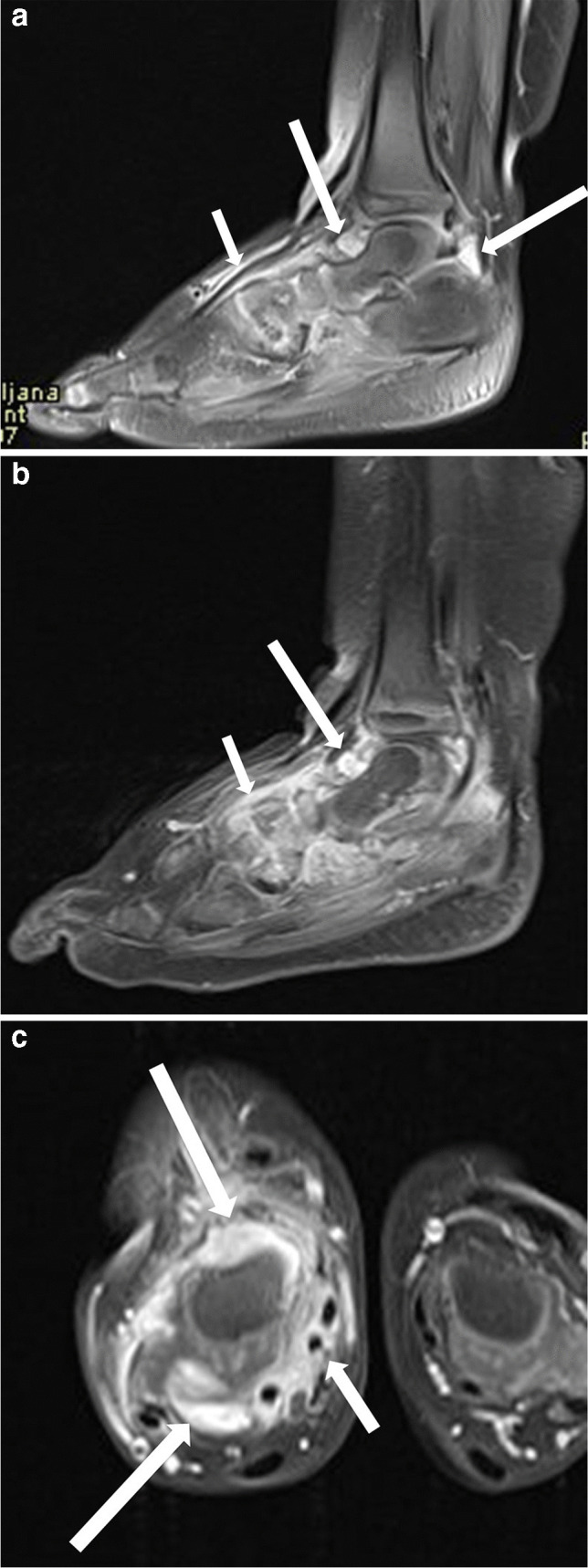
Magnetic resonance imaging of the right ankle in a 2-year-old girl with juvenile idiopathic arthritis (HLA B27 negative, anti-nuclear antibodies positive, same patient as in Fig. 5). **a** Sagittal proton-density-weighted fat suppressed image shows marked synovial proliferation and effusion of the anterior and posterior recesses of the ankle joint (*long arrows*) and around the extensor tendons (*short arrow*)—signs of synovitis and tenosynovitis. **b** Sagittal postcontrast T1-weighted fat-suppressed sequence shows marked synovial proliferation and enhancement of the anterior recess of the ankle joint (*long arrow*) and around the tarsal bones (*short arrow*)—signs of active synovitis. **c** Axial postcontrast T1-weighted fat-suppressed sequence shows marked synovial proliferation and enhancement of the anterior and posterior recesses of the ankle joint (*long arrows*) and around the flexor tendons (*short arrow*)—signs of active synovitis and tenosynovitis

**Fig. 7:**
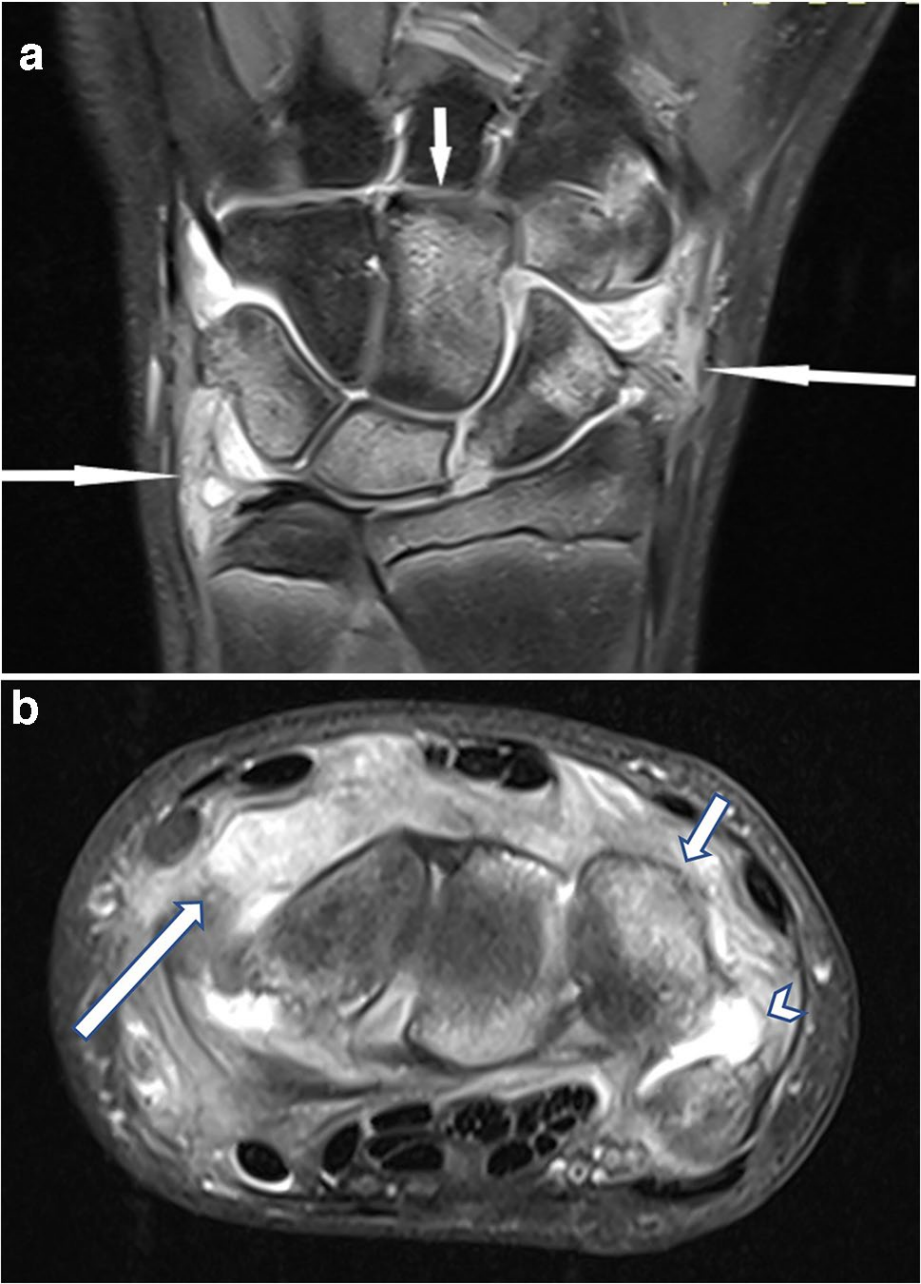
Magnetic resonance imaging of the right wrist in a 14-year-old boy with juvenile idiopathic arthritis with enthesitis (HLA B27 positive). Coronal (**a**) and axial (**b**) proton-density-weighted fat-suppressed images show marked synovial proliferation (*long arrows*), reactive edema of the carpal bones (*short arrows*), and a small joint effusion (*arrowhead* in **b**)—signs of synovitis

MRI is the most promising imaging technique for assessing the presence and extent of inflammation (synovial hypertrophy, joint effusion, soft tissue swelling), bone marrow changes, and cartilage status (Figs. 6 and 7). Furthermore, MRI can serve as a diagnostic tool for certain intra-articular pathologies that mimic JIA [81, 82].

As with all imaging modalities, the main criticisms of using MRI include the challenge of differentiating between pathological and physiological changes in bone marrow depending on the age and sex of the patient [83–91]. There is also a need for standardization, quantification, and validation of scoring systems to rigorously and consistently assess joint changes, in both cross-sectional and longitudinal studies. It is worth noting that efforts are currently underway to achieve this standardization [92].

The importance of MRI for monitoring inflammation and response to treatment has been confirmed. In 2015, the EULAR-PRES task force [6] and the European Society of Musculoskeletal Radiology (ESSR) Arthritis Subcommittee [93] published the indications for performing MRI for diagnosis, monitoring, and prediction, as well as MRI protocols for the most commonly affected joints in JIA; these have recently been updated by the ESSR and the European Society of Paediatric Radiology musculoskeletal imaging taskforce [19].

**Fig. 8:**
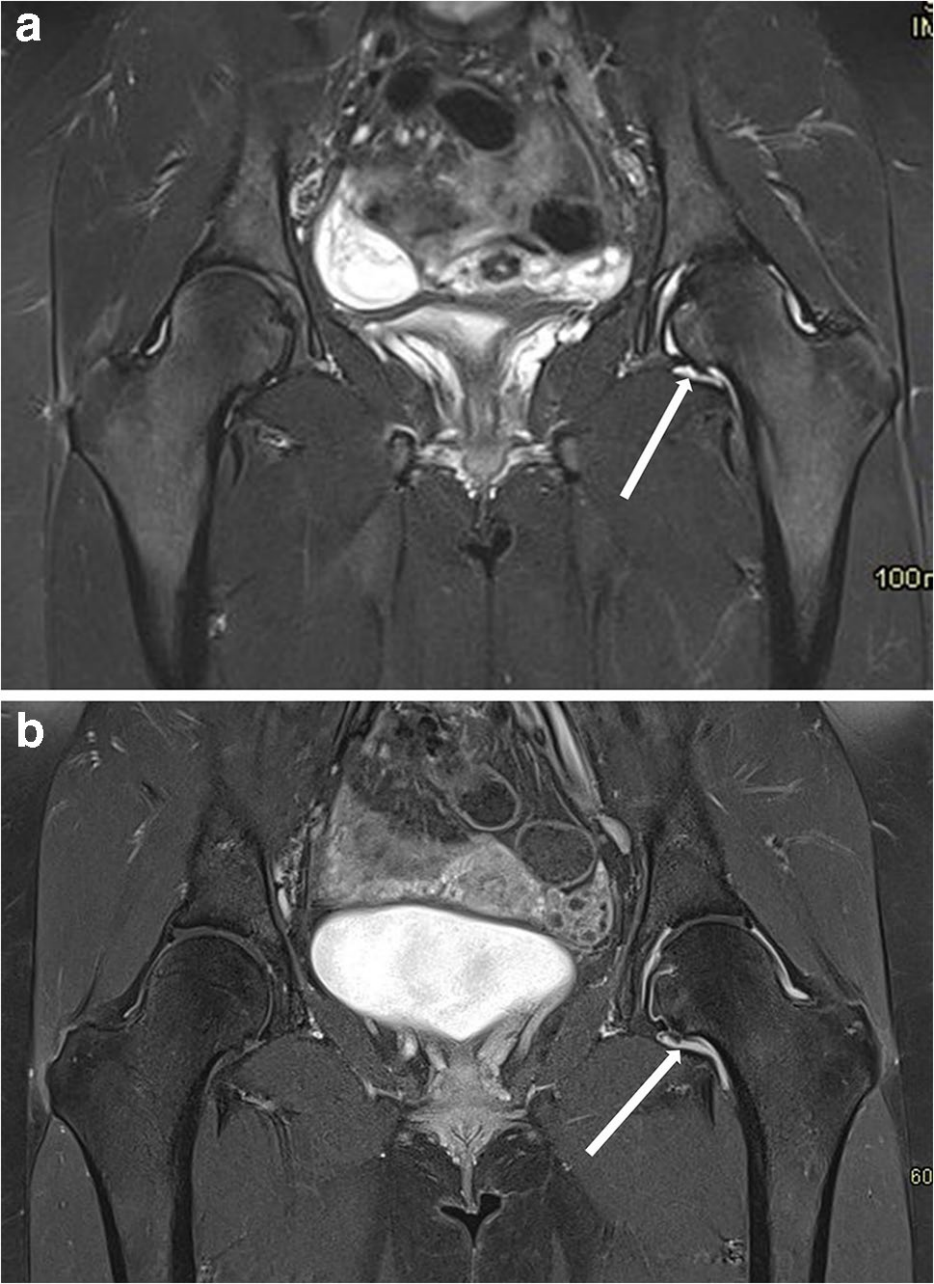
Coronal magnetic resonance imaging of the hips in a 15-year-old girl with psoriatic arthritis (HLA B27 negative, ANA positive). **a **T2 turbo inversion recovery image shows an effusion in the left hip joint (*arrow*); bone and cartilage are normal in appearance. **b** Postcontrast T1-weighted fat-suppressed image shows synovial enhancement on the left (*arrow*)—a sign of active synovitis

Emerging issues in MRI surveillance of JIA patients relate to the presence of subclinical synovitis as a predictor of disease flare-up (Fig. 8) [12]. Bone marrow edema is a questionable predictor of an unfavorable outcome, as it can be found in more than 50% of healthy children, as shown by the Norwegian group [87].

MRI has demonstrated greater sensitivity than US and CR in detecting bone erosions, even in the early stages of the disease [94–96]. In contrast, there are conflicting data regarding the detection of cartilage erosions, probably due to the lack of cartilage-specific sequences in MRI protocols for JIA [36].

Specifically, standardized MRI protocols and semi-quantitative classification systems have been developed to assess inflammation and osteochondral changes in the large and small joints of JIA patients. These are currently undergoing validation, including assessing their correlation with clinical disease activity [97–102]. The ability to identify a “target joint” that reflects the global burden of disease activity may be an optimal target [46, 97].

**Fig. 9:**
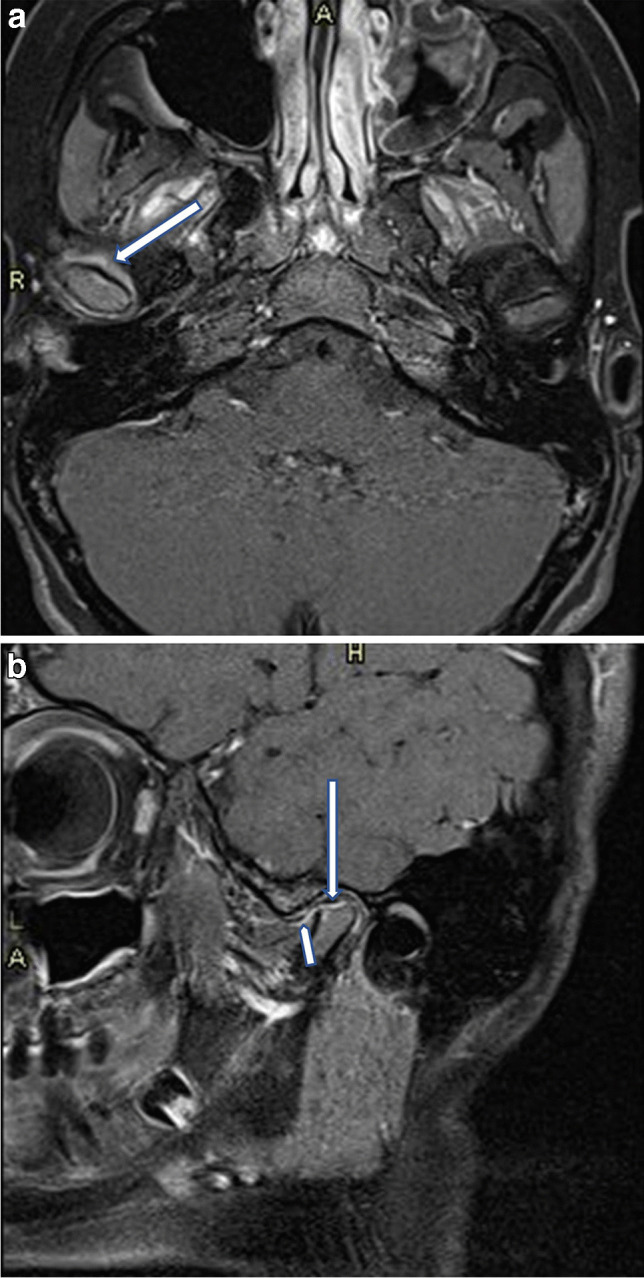
Postcontrast T1-weighted fat-suppressed magnetic resonance images of the temporomandibular joints in a 14-year-old girl with oligoarticular juvenile idiopathic arthritis (ANA positive). **a** Axial image shows synovial thickening and enhancement on the right (*arrow*)—a sign of active synovitis. **b** Sagittal image shows a flattened mandibular head (*long arrow*) and synovial thickening and enhancement on the right (*short arrow*)—signs of chronic bone changes and active synovitis

MRI is highly valuable for difficult-to-access joints like the TMJ and the axial skeleton, which are commonly affected in JIA. Early detection is essential to prevent functional issues, including mandibular condyle growth inhibition and micrognathia (Fig. 9) [98, 102–104]. Recently, a consensus MRI protocol for the examination of the TMJ has been developed by Inarejos Clemente et al. [20], describing the degree of normal and pathological findings using the currently available MRI scoring systems developed for JIA.

Contrast-enhanced MRI is the preferred method for identifying cervical spine involvement, a significant prognostic factor for JIA progression [21, 22]. It has demonstrated higher sensitivity than clinical examination, although cervical arthritis is often clinically silent [23].

MRI is valuable for monitoring disease progression, response to treatment, and evaluation of late changes and complications, including atlantoaxial instability, dens deformity, joint ankylosis, and spinal cord compression [105].

MRI is also the method of choice for the assessment of the SIJ.

Nevertheless, the ASAS criteria, commonly used in adults to evaluate both active inflammatory and structural lesions, may present challenges applied to children [42].

The OMERACT expert working group together with OMERACT-JAMRIS-SIJ is developing and evaluating a preliminary pediatric consensus scoring system of SIJMRI. This system assesses inflammation and structural changes in the SIJ of children, including erosion, sclerosis, fat lesion, and ankylosis considering growing bone and active bone marrow [10].

In addition, pelvic MRI in juvenile ankylosing spondylitis is also valuable for assessing enthesopathy at the tendon and fascial attachment sites and for coxofemoral joint involvement, which is often associated with sacroiliitis [13].

Whole-body MRI is a promising tool for detecting and monitoring inflammation involving the peripheral joints, the axial joints, and the entheses in rheumatological diseases such as spondyloarthropathies [106–108]. On the other hand, there are no clear guidelines for the standardized detection, interpretation, and quantification of JIA on whole-body MRI. Moreover, MRI is still not widely available in clinical practice due to limitations of cost, access, and relatively long acquisition time, requiring sedation or general anesthesia in young children.

In an effort to reduce the use of gadolinium-based contrast agents, Barendregt et al. [109] conducted interesting research on the potential use of diffusion-weighted imaging as an alternative for the assessment of synovial inflammation.

In summary, MRI is an excellent method for monitoring disease activity in response to treatment, especially in difficult-to-access joints such as the axial skeleton (spine and SIJs) and TMJs. It is also very sensitive in detecting subclinical arthritis, the importance of which needs to be further assessed. The importance of bone marrow edema as a potential bad outcome predictor also needs to be further investigated.

### Advantages and disadvantages

The advantages and disadvantages of imaging modalities in JIA are systematically presented in Table 3 (XR), Table 4 (US), and Table 5 (MRI).

**Table 3:** Advantages and disadvantages of conventional radiology in juvenile idiopathic arthritis

Advantages	Disadvantages
• Rapidity of performance • Applicability to all joints • Demonstration of joint space narrowing, disturbances of bone growth, and maturation • Detection of bone erosions • Validated scoring methods in children • Suitable for longitudinal evaluation of damage progression • Low cost • Widespread availability	• Exposure to ionizing radiation • Inability to directly visualize cartilage and soft tissue inflammation • Late detection of bone erosions and joint space narrowing (normal in early stage) • Projectional superimposition • Limited use in some sites (TMJ involvement overlooked) • Misinterpretation of anatomic variation as bone erosion (wrist joint)

*TMJ* temporomandibular joint

**Table 4:** Advantages and disadvantages of ultrasound imaging in juvenile idiopathic arthritis

Advantages	Disadvantages
• Non-invasiveness • Relatively inexpensive • Lack of exposure to ionizing radiation • No need to sedate children • Possibility to assess several joint regions in a single scanning session • Capability of dynamic and real-time assessment • Potential guidance for corticosteroid injections in joints, tendon sheaths, or synovial bursas • Portability • Rapidity of performance • Ease of repeatability • Visualization of soft tissue inflammation	• Operator dependency • Reliability dependent on sensitivity of US equipment • Not all joints assessable • Inability to assess the whole joint space • Relatively small field of view • Acoustic shadowing from overlying bones • Difficult to carry out in case of joint functional limitation and/or pain • Lack of validated scoring systems to quantify US abnormalities • Difficult to standardize and centralize for clinical trials

*US* ultrasound

**Table 5:** Advantages and disadvantages of magnetic resonance imaging in juvenile idiopathic arthritis

Advantages	Disadvantages
• Lack of exposure to ionizing radiation • Multiplanar tomographical imaging • Ability to assess the whole joint space • Demonstration of soft tissue inflammation • Direct visualization of cartilage • Early detection of bone erosions • Visualization of bone marrow edema • High tissue contrast • Suitable for assessment of axial skeleton and temporomandibular joints	• Intravenous contrast agent often required • Possible allergic reaction to contrast agents • General anesthesia required in younger children • Long examination time • Evaluation limited to one target joint • Reliability, standardization, and validation in children under investigation • High cost • Variable availability worldwide

## Conclusion

This article provides a concise overview of the clinical utility of different imaging modalities in patients with JIA. Imaging plays an important role in confirming the diagnosis of JIA, assessing joint and bone involvement, and in tracking disease activity and treatment response. In addition, imaging can potentially predict poor prognosis by detecting subclinical inflammation or structural damage, even in cases of clinically inactive disease. Nonetheless, it is crucial to use these imaging modalities judiciously, either to confirm or complement findings from physical examination of the musculoskeletal system.

## Data Availability

The images in the manuscript are available from the authors upon reasonable request. No other datasets were generated for this manuscript.
